# Frequencies of CD33+CD11b+HLA-DR–CD14–CD66b+ and CD33+CD11b+HLA-DR–CD14+CD66b– Cells in Peripheral Blood as Severity Immune Biomarkers in COVID-19

**DOI:** 10.3389/fmed.2020.580677

**Published:** 2020-10-14

**Authors:** Ricardo Wesley Alberca, Milena Mary de Souza Andrade, Anna Cláudia Calvielli Castelo Branco, Anna Julia Pietrobon, Nátalli Zanete Pereira, Iara Grigoletto Fernandes, Luana de Mendonça Oliveira, Franciane Mouradian Emidio Teixeira, Danielle Rosa Beserra, Emily Araujo de Oliveira, Sarah Cristina Gozzi-Silva, Yasmim Álefe Leuzzi Ramos, Cyro Alves de Brito, Marcelo Arnone, Raquel Leao Orfali, Valeria Aoki, Alberto Jose da Silva Duarte, Maria Notomi Sato

**Affiliations:** ^1^Laboratorio de Dermatologia e Imunodeficiencias (LIM-56), Departamento de Dermatologia, Faculdade de Medicina FMUSP, Universidade de São Paulo, São Paulo, Brazil; ^2^Departamento de Imunologia, Instituto de Ciências Biomédicas, Universidade de São Paulo, São Paulo, Brazil; ^3^Technical Division of Medical Biology, Immunology Center, Adolfo Lutz Institute, São Paulo, Brazil; ^4^Departamento de Dermatologia, Faculdade de Medicina FMUSP, Universidade de São Paulo, São Paulo, Brazil; ^5^Departamento de Patologia, Faculdade de Medicina FMUSP, Universidade de São Paulo, São Paulo, Brazil

**Keywords:** SARS-CoV-2, infection, COVID-19, biomarker, severity

## Abstract

Common clinical features of patients with Coronavirus disease-2019 (COVID-19) vary from fever, to acute severe respiratory distress syndrome. Several laboratory parameters are reported as indicators of COVID-19 severity. We hereby describe the possible novel severity biomarkers for COVID-19, CD11b+CD33+HLA-DR-CD14+ cells and CD11b+CD33+HLA-DR-CD66b+ cells.

## Introduction

Severe acute respiratory syndrome coronavirus 2 (SARS-CoV-2) is the etiologic agent of COVID-19. COVID-19 has already led to massive infection and deaths worldwide and was declared a pandemic by the World Health Organization.

Common clinical features of patients with COVID-19 vary from fever, cough, dyspnea (among others) to acute severe respiratory distress syndrome (ARDS), and shock. Reports of fatal outcomes in hospitalized patients vary from 6.2 to 21.5% (1).

Laboratory parameters such as neutrophil-to-lymphocyte ratio (2, 3), C-reactive protein (CRP), interleukin (IL)-6 and dimer-D levels (4) are reported as indicators of COVID-19 severity when associated to clinical features of the infection. COVID-19 patients hospitalized at intensive care unit (ICU) exhibit augmented levels of inflammatory cytokines and infection biomarkers, such as interleukin (IL)-1B, IL-7, IL-8, IL-9, IL-10, interferon (IFN)-γ, interferon gamma-inducible protein (IP)-10, monocyte chemoattractant protein (MCP)-1, macrophage inflammatory protein (MIP)1A, MIP1B, platelet-derived growth factor (PDGF), tumor necrosis factor-alpha (TNFα), and vascular endothelial growth factor (VEGF). Furthermore, IL-2, IL-7, IL-10, IP-10, MCP1, MIP1A, and TNF are increased in ICU patients in comparison with non-ICU patients (5). Of note, there are reports on the presence of lymphocytopenia (6) and decreased levels of suppressor, regulatory and memory T cells in those patients with severe COVID-19 infection (6).

During inflammation and infection, many different cells expand (7), including Monocytic Myeloid-Derived Suppressor Cells (M-MDSCs) characterized by the surface markers CD33+CD11b+HLA-DR-CD14+ and the low-density polymorphonuclear MDSCs (PMN-MDSCs) characterized by the surface markers CD33+CD11b+HLA-DR-CD66b+ (8). These myeloid cells possess suppressive effects on innate and adaptive immune responses, modulating cytokine, reactive oxygen and nitrogen species (7).

In this context, we investigated, with a whole blood assay, the frequency of CD33+CD11b+HLA-DR-CD14+ cells and CD33+CD11b+HLA-DR-CD66b+ cells (containing high-density neutrophils as well as low-density PMN-MDSCs) in patients recently infected with SARS-CoV-2.

## Methods

We investigated the presence of CD33+CD11b+HLA-DR-CD14+ and CD33+CD11b+HLA-DR-CD66b+ cells in the peripheral blood of 104 patients infected with COVID-19, 62 males and 42 females. The diagnosis of COVID-19 was confirmed by the detection of SARS-CoV-2 RNA by reverse-transcriptase polymerase chain reaction (RT-PCR), and patients tested negative for respiratory syncytial virus (RSV) and influenza. The study was approved by the Ethics Committee of Hospital das Clínicas da Faculdade de Medicina da Universidade de São Paulo—HCFMUSP (no. 30800520.7.0000.0068-2020) and was carried out in conformity with the 2013 revision of the Declaration of Helsinki. Healthy controls (HC), without SARS-CoV-2, RSV or influenza infection, were recruited for flow cytometry assays (*n* = 16, 7 males, 9 females, median age 49.7 years).

COVID-19 patients were hospitalized and characterized as follows: 49 were admitted into ICU and needed assisted mechanical ventilation, and 55 were moderate and remained at the general ward (GW).

During hospitalization, COVID-19 patients received systemic treatment. All patients received antibiotics and anticoagulants, 45/104 systemic corticosteroids, 39/104 antivirals. EDTA plasma samples were obtained from a single venipuncture. Laboratory analysis was performed at the Central Laboratory of Hospital das Clinicas, Faculdade de Medicina da Universidade de São Paulo (Divisão de Laboratório Central—HC FMUSP), and included: complete blood counts (CBC), coagulogram, liver enzymes (alanine aminotransferase—ALT and aspartate aminotransferase—AST), alkaline phosphatase (AP), bilirubin, urea, creatinine, glucose, sodium, potassium, lactate dehydrogenase, magnesium, phosphorus, total proteins (albumin) and fractions immunoglobulins, CRP, ferritin, pH, pO2, pCO2, D-dimer, and erythrocyte sedimentation rate in arterial blood collected in K2EDTA tubes.

**Figure 1:**
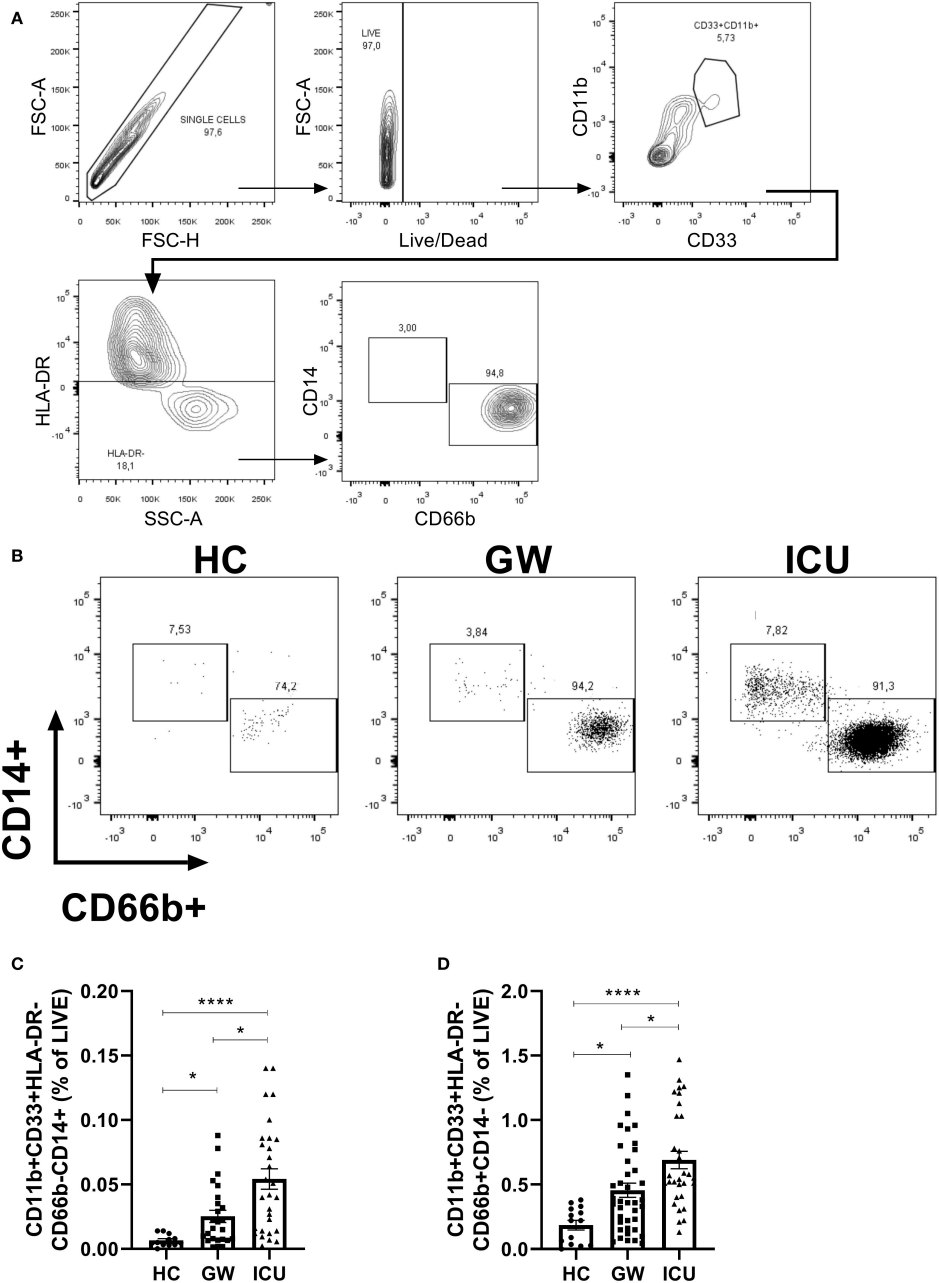
Frequencies of CD11b+CD33+HLA-DR-CD14+ cells or CD11b+CD33+HLA-DR-CD66b cells in peripheral blood of HC, GW, and ICU COVID-19 patients. **(A)** Gate strategy used for flow cytometry: single cells, live, CD11b+CD33+HLA-DR-CD14+ or CD66b+; **(B)** Representative plots for HC, GW, and ICU; **(C)** Frequency of CD11b+CD33+HLA-DR-CD14+ and **(D)** CD11b+CD33+HLA-DR-CD66b in the peripheral blood of HC, GW, and ICU. Kruskal-Wallis test with Dunn's post-test. *p < 0.05, ****p < 0.0001.

Flow cytometry analysis was performed using 0.1 mL of whole blood in K2EDTA collection tubes (9). Samples from 104 patients and 16 HC were incubated with a fixable viability probe (Live/Dead, Life technologies) for 20 min at 4°C, for cell viability analysis in the flow cytometer. Subsequently, were washed and then incubated for 60 min at 4°C, with the antibodies mix containing: anti-CD11b PE (ExBio, clone MEM-78), anti-CD33 PERCP (ExBio, clone MEM-174), anti-CD14 FITC (BD-Bioscences, clone M5E2), anti-CD66b BV421 (BD-Biosciences, clone G10F5), anti-HLA-DR V500 (BD-Biosciences, clone G46-6). Data are shown as median values and standard error (SEM). The between-group differences were analyzed using the Mann Whitney test for independent samples for clinical features in Table 1, and Kruskal-Wallis test with Dunn's post-test for Flow cytometry analysis (Figure 1) (*P* < 0.05).

**Table 1:** Patients' characteristics by the severity of the disease.

	**General ward (*****N*** **=** **55)**		**Intensive care (*****N*** **=** **49)**			
	**MEAN**	**SEM**	**MEAN**	**SEM**	**Reference numbers**	***p*-value**
**Male/Female**	28/27	34/15				
**Age (years)**	54.42	1.807	55.37	1.797	-	0.8364
**Gasometry**						
pH	7.383	0.01555	7.370	0.01121	7.35–7.45	0.5128
pO2 (mmHg)	**51.72**	**5.025**	63.48^*^	**3.338**	**30–50**	**0.0382**
pCO2 (mmHg)	**40.26**	**1.676**	45.19^*^	**1.123**	**38–50**	**0.0167**
**Laboratory Data**						
Erythrocytes (million/mm^3^)	3.834	0.1174	3.619	0.1264	3.9–5.6	0.1696
Hemoglobin (g/dL)	10.88	0.3311	10.38	0.3504	11.5–15.5	0.2815
Hematocrit (%)	32.53	0.9594	31.30	0.9825	36–48	0.3685
Mean corpuscular volume (fL)	**84.68**	**0.8690**	**87.30**	**0.9571**	**80–95**	**0.0226**
Mean corpuscular hemoglobin (pg)	28.29	0.3241	28.86	0.3412	27–34	0.1775
Mean corpuscular hemoglobin concentration (g/dL)	33.44	0.1704	33.08	0.1687	30–35	0.1876
Red cell distribution width–coefficient of Variation (%)	14.30	0.2338	14.99	0.3259	11.7–14.4	0.2385
Leukocytes (×10^3^/mm^3^)	**8.470**	**0.7366**	12.94^****^	**0.9811**	**04–11**	**0.0001**
Neutrophils (×10^3^/mm^3^)	**6.073**	**0.5981**	10.74^****^	**0.8717**	**2.5–7.5**	**0.0001**
Eosinophils (×10^3^/mm^3^)	0.1256	0.0211	0.1825	0.0415	0.04–0.4	0.8356
Basophils (×10^3^/mm^3^)	0.08870	0.0523	0.04500	0.0096	0–0.1	0.2765
Lymphocytes (×10^3^/mm^3^)	1.365	0.1179	1.163	0.1090	1.5–3.5	0.2957
Monocytes (×10^3^/mm^3^)	0.7572	0.0981	0.7406	0.0840	0.2–0.8	0.9461
Ratio Neutrophil-to-Lymphocyte	**5.413**	**0.5880**	17.39^****^	**3.740**	**4–11**	**0.0001**
Alanine aminotransferase (U/L)	37.26	5.238	53.69	8.163	<41	0.2414
Asparate aminotransferase (U/L)	32.67	2.369	47.08	5.550	<37	0.1659
Alkaline phosphatase (U/L)	**75.5**	**14.58**	**136.9**	**27.20**	**40–129**	**0.0150**
Total bllirubin (mg/dL)	**0.3007**	**0.0515**	**0.5714**	**0.0950**	**0.2–1.0**	**0.0363**
Direct bilirubin (mg/dL)	**0.2050**	**0.03151**	**0.4652**	**0.08875**	** <0.3**	**0.0113**
Indirect bilirubin (mg/dL)	0.095	0.0253	0.08259	0.01074	0.1–0.6	0.8754
Glutamyl transferase gamma (U/L)	**115.3**	**43.86**	**320.8**	**107.6**	**8–61**	**0.0388**
Ionic calcium (mg/dL)	5.035	0.2257	4.759	0.0635	4.49–5.29	0.7189
Chlorine (mEq/L)	100	1.181	100.6	0.8911	98–107	0.6872
Creatinine (mg/dL)	**1.909**	**0.3148**	2.427^*^	**0.2621**	**0.7–1.2**	**0.0121**
Glicose (mg/dL)	150.5	29.59	244.5	28.09	70–100	0.1830
Potassium (mEq/L)	4.368	0.0822	4.560	0.1117	3.5–5.0	0.2737
Lactate dehydrogenase (U/L)	**362.9**	**56.68**	507^*^	**36.75**	**135–225**	**0.0121**
Magnesium (mg/dL)	**1.978**	**0.0618**	2.241^**^	**0.0614**	**1.58–2.55**	**0.0023**
Sodium (mEq/L)	137.6	0.8027	138.5	0.8747	135–145	0.6411
C-reactive protein (mg/L)	**74.46**	**13.98**	128.7^*^	**18.85**	** <5.0**	**0.0408**
Phosphorus (mg/dL)	3.635	0.3702	4.095	0.3768	2.7–4.5	0.2342
Total Protein and fractions (g/dL)	5.425	0.2955	5.885	0.2703	6.6–8.7	0.4231
Albumin (g/dL)	3.025	0.2056	2.823	0.1297	3.4–4.8	0.5992
Urea (mg/dL)	**62.08**	**6.085**	99.15^**^	**10.15**	**10–50**	**0.0016**
Platelets (×10^3^/mm^3^)	282.2	20.19	272.2	20.87	150–400	0.6375
D-dimer (ng/mL)	**1443**	**228.8**	**7165**	**2019**	** <500**	**0.0012**
Prothrombin time (s)	15.68	1.408	13.89	0.3638	9.4–12.5	0.4424
Activated partial thromboplastin time (s)	36.02	4.044	40.74	2.927	25.1–36.5	0.6071
**Comorbidities/No Comorbidities**						
Diabetes mellitus/Metabolic syndrome	29/26		27/22			
Systemic Arterial Hypertension	29/26		27/22			
Heart disease	10/45		20/29			
Renal disease	15/40		7/42			
Human immunodeficiency virus	1/54		2/47			
Associated malignant neoplasia	7/48		3/46			

* *P < 0.05*,

** *P < 0.01*,

*** P < 0.001 and

**** *p < 0.0001 were considered statistically significance*.

*P-values indicate differences between general ward and intensive care unit patients. Reference values from Divisão de Laboratorio Central do HC/FMUSP. Bold indicates outside the reference interval*.

## Results

Table 1 depicts the clinical features of COVID-19 patients. Both GW and ICU patients showed significant reduction in erythrocytes, hemoglobin, hematocrit, total protein, albumin in relation to reference levels. Higher levels in gamma-glutamyl transferase, creatinine, lactate dehydrogenase, C-reactive protein (CRP), urea, D-dimer, prothrombin time both GW and ICU patients were detected. Mean levels of pO2. Red cell distribution width–coefficient of variation (RDW-CV), leukocytes, neutrophils, neutrophil-to-lymphocyte ratio, alanine aminotransferase, aspartate aminotransferase, alkaline phosphatase, direct bilirubin, creatinine, potassium, lactate dehydrogenase and activated partial thromboplastin time were increased in ICU patients, but not GW patients, in comparison to reference values (Table 1).

ICU patients, when compared to GW subjects, exhibited increased pCO2 (*p* = 0.0382), pCO2 (*p* = 0.0167), mean corpuscular volume (*p* = 0.0226), enhanced leukocytes (*p* = 0.0001), neutrophils (*p* = 0.0001), neutrophil-to-leukocyte ratio (*p* = 0.0001), and augmented levels of alkaline phosphatase (*p* = 0.0150), total bilirubin (*p* = 0.0363), direct bilirubin (*p* = 0.0113), glutamyl transferase gamma (*p* = 0.0388), creatinine (*p* = 0.0121), lactate dehydrogenase (*p* = 0.0121), CRP (*p* = 0.0408), urea (*p* = 0.0016) and D-dimer (*p* = 0.0012) (Table 1).

Such laboratory parameters reinforce the need for biomarkers such as neutrophil-to-lymphocyte ratio, urea and d-dimer as a predictive factor of disease outcome (2, 4). Moreover, lactate dehydrogenase, a well-known marker for persistent viral infections may be of relevance as a potential biomarker for COVID-19 (10). Several manuscripts have highlighted the influence of comorbities in COVID-19 such as old age, systemic arterial hypertension, pregnancy and obesity (11–14).

Previous reports verified an immune dysregulation in circulating immune cells in the blood of COVID-19 patients, with a significant increase in neutrophil-to-lymphocyte ratio in COVID-19, and a further increase in severe COVID-19 (6). Here, we verified an increased percentage of CD11b+ CD33+ HLA- in the blood ICU patients in comparison with GW patients with gate strategy as depicted in Figure 1A. This population, scarce in blood samples of HC (Figure 1B), could be split into CD14+ cells and CD66b+ populations (Figure 1B).

Quantifications of the frequency of CD33+CD11b+HLA-CD14+ and CD33+CD11b+HLA-DR-CD66b+ populations, both in relation to the total of live cells, are shown in (Figures 1C,D). We verified an increased percentage of CD33+CD11b+HLA-DR-CD14+ cells in the blood of GW patients (mean = 0.025, SEM = 0.004; median = 0.016, 25% percentile = 0.007, 75% percentile = 0.037; Lower 95% CI = 0.015, Upper 95% CI = 0.035) in comparison with HC individuals (mean = 0.006, SEM = 0.001; median = 0.004, 25% percentile = 0.003, 75% percentile = 0.010; Lower 95% CI = 0.003, Upper 95% CI = 0.009) (*p* = 0.0471). We also verified an increase in CD33+CD11b+HLA-DR-CD14+ cells in the blood of ICU patients (mean = 0.054, SEM = 0.007; median = 0.042, 25% percentile = 0.014, 75% percentile = 0.085; Lower 95% CI = 0.038, Upper 95% CI = 0.070) in comparison with HC individuals (*p* < 0.0001) and GW patients (*p* = 0.0388) (Figure 1C).

The frequency of CD33+CD11b+HLA-DR-CD66b+ cells were also increased GW (mean = 0.45, SEM = 0.05; median = 0.36, 25% percentile = 0.16, 75% percentile = 0.68; Lower 95% CI = 0.344, Upper 95% CI = 0.566) patients in comparison with HC individuals (mean = 0.18, SEM = 0.03; median = 0.22, 25% percentile = 0.03, 75% percentile = 0.31; Lower 95% CI = 0.105, Upper 95% CI = 0.266) (*p* = 0.0178). Again, we also verified an increase in CD33+CD11b+HLA-DR-CD66b+ cells in the blood of ICU patients (mean = 0.69, SEM = 0.06; median = 0.55, 25% percentile = 0.41, 75% percentile = 1.03; Lower 95% CI = 0.552, Upper 95% CI = 0.827) in comparison with HC individuals (*p* < 0.0001) and GW patients (*p* = 0.0175) (Figure 1D).

## Discussion

Overall, these findings suggest a correlation between the quantification of CD33+CD11b+HLA-DR-CD14+ cells and CD33+CD11b+HLA-DR-CD66b+ cells and the severity of COVID-19. These cells have been previously characterized as myeloid derived suppressor (MDSC) cells by this cytometry panel (8), but further investigations about their suppressor's activities are necessary to assure this designation. Nevertheless, these cells may elicit a suppressor activity in adaptive and innate immunity, disrupting anti-viral immune response, via regulation of T cell and NK cell activation, the polarization of macrophages and immune cell trafficking (15). These cells play an important role in the suppression of immune response in chronic inflammatory conditions such as chronic infections, trauma and cancer (16). This is a pioneer study, which correlates the frequency of both CD33+CD11b+HLA-DR-CD14+ cells and CD33+CD11b+HLA-DR-CD66b+ cells to COVID-19 severity, as previous reports only highlighter the expansion of polymorphonuclear MDSC (17). Mononuclear MDSC and polymorphonuclear MDSC increase is usually described in chronic inflammation and infection (7), and overall, COVID-19 patients have a history of recent infection (minimum of three days). Due to the increase in inflammatory markers in COVID-19 patients, mononuclear MDSC and polymorphonuclear MDSC may indicate a regulatory mechanism to modulate inflammation, since the expansion of MDSC can be mediated by cytokines such as IL-6, an abundant cytokine in COVID-19 (7, 15). Another possible explanation is that MDSC curbs the anti-viral immune response, leading to an increase in the viral load and consequently aggravating SARS-CoV-2 infection (18). MDSCs classically belong to a group of cells that resemble macrophages and neutrophils, not only in morphology but also in phenotype; such features may be a barrier to identify and purify those cells. It is possible to assume that the population of CD33+CD11b+HLA-DR-CD14+ are monocytic MDSCs, but the CD33+CD11b+HLA-DR-CD66b+ cells in our study may contain both high-density neutrophils as well as the low-density PMN-MDSCs because of the use of whole blood (8), therefore further investigations are needed to state these cells as polymorphonuclear MDSC and their suppressive activity. In this report, we utilized a fast, simple and certified flow cytometry panel (8) that could be help to identify these biomarkers in COVID-19 patients.

## Conclusion

Enhanced CD33+CD11b+HLA-DR–CD14–CD66b+ and CD33+CD11b+HLA-DR–CD14+CD66b– cells in the blood of ICU patients, classified as severe COVID patients, could represent not only a predictor of prognosis for COVID-19, but also specific therapy targets for treating this virus infection in the near future.

## Data Availability Statement

All datasets generated for this study are included in the article/supplementary material.

## Ethics Statement

This studies involving human participants were reviewed and approved by Ethics Committee of Hospital das Clínicas da Faculdade de Medicina da Universidade de São Paulo—HCFMUSP (no. 30800520.7.0000.0068-2020). The patients/participants provided their written informed consent to participate in this study.

## Author Contributions

RA and MS: conception, performed experiments, analysis, write, and review. MAn, AB, AP, NP, IF, LO, FT, DB, EO, SG-S, YR, and CdB: performed experiments and review. MAr, RO, VA, and AD: patient care and review. All authors contributed to the article and approved the submitted version.

## Conflict of Interest

The authors declare that the research was conducted in the absence of any commercial or financial relationships that could be construed as a potential conflict of interest.
